# Structures of Pb-BHA Complexes Adsorbed on Scheelite Surface

**DOI:** 10.3389/fchem.2019.00645

**Published:** 2019-09-24

**Authors:** Zhao Wei, Wenjuan Sun, Yuehua Hu, Haisheng Han, Wei Sun, Ruolin Wang, Yangge Zhu, Bicheng Li, Zhenguo Song

**Affiliations:** ^1^School of Minerals Processing and Bioengineering, Central South University, Changsha, China; ^2^Key Laboratory of Hunan Province for Clean and Efficient Utilization of Strategic Calcium-Containing Mineral Resources, Central South University, Changsha, China; ^3^B.Grimm Technology Group, Beijing, China

**Keywords:** Pb-BHA complexes, adsorption capacity, XPS, TOF-SIMS, structure fragment

## Abstract

Previous studies have shown that Pb-BHA complexes (lead complexes of benzohydroxamic acid) have better collecting ability and can be used in flotation experiments with BHA acting as a collector and lead ions acting as activators. However, the structures of Pb-BHA complexes adsorbed on a mineral surface remain unclear. In this work, the adsorption behavior of Pb-BHA complexes on the scheelite surface was studied by flotation experiments and adsorption capacity measurements, and the structures of the adsorbed Pb-BHA complexes were determined using X-ray photoelectron spectroscopy (XPS) and time-of-flight secondary ion mass spectrometry (TOF-SIMS). The adsorption capacity results showed that more BHA was adsorbed on the scheelite surface in Pb-BHA flotation, and the XPS and TOF-SIMS analysis showed that the species of Pb-BHA complexes adsorbed on the scheelite surface were similar in activation flotation and Pb-BHA flotation. Therefore, the different contents of the complexes on the scheelite surface were responsible for the flotation behavior. XPS and TOF-SIMS showed that BHA combined with lead ions to form complexes with different structures, such as five- and four-membered ring structures. Structure fragment inference based on the measurements indicated that lead ions formed monomer complexes with two BHAs, and that lead hydroxide polymers with a certain degree of polymerization bonded with oxygen atoms in the complexes. The Pb-BHA complexes combine with oxygen atoms on the scheelite surface to form an adsorbate, rendering the surface hydrophobic.

## Introduction

Flotation is a widely used process for material separation, based on differences in the surface hydrophobicity and hydrophilicity of minerals. The separation of scheelite from other calcium-containing minerals such as fluorite and calcite has been difficult owing to their similar surface properties (Li and Gao, 2017; Gao et al., 2018, 2019; Jiang et al., 2018). However, previous studies have shown that Pb-BHA complexes have good collection ability and high selectivity in scheelite flotation. Their use in industrial applications solved the problem of the separation of scheelite from other calcium-bearing minerals, replacing the Petrov process to some extent (Han et al., 2017a; Wei et al., 2018; Yue et al., 2018). The better collection ability and selectivity of Pb-BHA complexes in calcium mineral flotation was attributed to be the unique molecular structure of these complexes. However, the structure of Pb-BHA complexes adsorbed on the scheelite surface remained unclear. Previous studies have shown that the different molar ratio of Pb/BHA will influence the flotation performance of Pb-BHA complexes collector (Han et al., 2017a), indicating the structure of complexes will influence their industrial applications. Therefore, molecular-level understanding of the Pb-BHA complexes is required to improve their industrial applications.

Few research studies have focused on the formation and configuration of Pb-BHA complexes in solution or on a mineral surface (Tian et al., 2017, 2018a,b; Yue et al., 2017; He et al., 2018b; Hu et al., 2018; Cao et al., 2019). In particular, solution-chemistry-based studies of the respective components of Pb-BHA complexes in cassiterite flotation (Tian et al., 2017, 2018a,b; Cao et al., 2019) showed that lead ions and their hydroxide ions form Pb(H_2_O) and Pb(OH)(H_2_O) with water and then react with the BHA anion to produce HO-Pb-BHA complexes. He et al. (2018a,b) characterized the structure of Pb-BHA complexes in aqueous solutions through theoretical calculations and quantum chemical simulations; the results showed that the Pb-BHA complexes existed mainly in the form of Pb-(BHA)_n = 1, 2_ at various solution pH values, and first-principles calculations indicated that Pb(BHA)^+^ is the most stable structure. Both studies concluded that the Pb-BHA complexes in the flotation did not consist of a single structure and proposed that Pb-BHA complexes with multiple structures acted simultaneously on the mineral surface. However, these studies only analyzed the structure of Pb-BHA complexes in theory by using approaches such as solution chemistry, theoretical calculations, and simulations. Although such studies are very helpful in understanding the structure of complexes, experimental research was lacking, which is necessary to investigate the true structure of Pb-BHA complexes adsorbed on mineral surfaces. In addition, these studies usually considered the structure of Pb-BHA complexes in solution, but the structure and species composition of Pb-BHA complexes adsorbed on a mineral surface or the solid–liquid interface may be different (Rao, 2004; Bulatovic, 2015; Kupka and Rudolph, 2018), this was not studied in detail. Moreover, the differences between structures of complexes formed by lead ions and BHA in activation flotation and Pb-BHA complex flotation were not considered, although this is crucial for a better understanding of Pb-BHA complex flotation.

In this study, experimental research was conducted to investigate the structures of Pb-BHA complexes adsorbed on a scheelite surface. The adsorption behavior of Pb-BHA complexes on the scheelite surface was studied by flotation experiments and adsorption capacity measurements, and their structures were examined using X-ray photoelectron spectroscopy (XPS) and time-of-flight secondary ion mass spectrometry (TOF-SIMS), providing an in-depth understanding of the adsorption mechanism of Pb-BHA complexes on a mineral surface.

## Materials and Methods

### Materials and Reagents

High-purity scheelite samples were obtained from Shizhuyuan Mine, Hunan, China. X-ray diffraction patterns and X-ray fluorescence data confirmed that the purity of the scheelite samples was >97%. A fine fraction (~74 μm) was used for the experiments. Analytical grade BHA and lead nitrate were purchased from Tianjin Guangfu Fine Chemical Co. Ltd. (Tianjin, China), and the pH was adjusted with NaOH or HCl.

The Pb-BHA complexes used for the flotation tests and adsorption measurements were freshly prepared; solutions of lead nitrate and BHA were mixed in a beaker at the desired mole ratio, mixed evenly, and allowed to stand until use. The powdered Pb-BHA complexes precipitates used for XPS, X-Ray Diffraction (XRD), Fourier Transform Infrared Spectroscopy (FTIR), and Thermogravimetric analysis (TGA) measurements were prepared in solution and dried in a vacuum oven at 40°C, which the molar ratio of Pb/BHA in complexes were 1:1. The characterization of the Pb-BHA complexes as shown in Supporting Information, including XRD, FTIR, and TGA.

### Flotation Test

The flotation tests were carried out in an XFG flotation machine with a 40 mL plexiglass cell at an impeller speed of 1,900 rpm. For each test, 2.0 g of the mineral sample was dispersed into 40 mL of deionized water in the cell and the flotation pulp was mixed for 1 min. The experimental procedure was as described previously (Han et al., 2017a; Wei et al., 2019). Recovery was calculated according to Equation (1), where R is the recovery, M_1_ is the mass of the concentrate, and M_2_ is the mass of the tailings. All the flotation experiments were conducted more than three times to ensure that the standard deviation of the experimental results was within 3%. The average value of all measurements conforming to this standard deviation was taken as the final experimental data.

(1) $$\begin{array}{r} \text{R}=\frac{{\text{M}}_{\text{1}}}{{\text{M}}_{1}+{\text{M}}_{2}}\times 100 \end{array}$$

### Adsorption Capacity Measurements

For each test, 2.0 g of scheelite powder was mixed in desired amounts of flotation reagents in the flotation cell, and deionized water was added to make the total volume 40 mL. The process was the same as that used in the flotation experiments. Afterwards, the suspension was centrifuged for 30 min at a speed of 9,000 rpm, and the residual concentration of BHA in the supernatant was determined using a total organic carbon analyzer (TOC-L CPH/CN, Shimadzu Corporation). The amount of BHA adsorbed on the mineral surface was calculated according to Equation (2):

(2) $$\begin{array}{r} \Gamma =\left(C_{0}-C\right)V/m \end{array}$$

where Γ is the amount of lead ions or BHA adsorbed on the mineral surface (mol/g), *C*_0_ and *C* are the initial and residual concentrations (mol/L) of lead ions or BHA, respectively, *V* is the volume of the solution (L), and *m* is the mass (g) of particles per sample.

### X-Ray Photoelectron Spectra Measurements

XPS measurements were conducted using a Thermo Fisher Scientific K-Alpha 1063 X system. The −74 μm fractions of pure scheelite samples (2.0 g) were conditioned with the appropriate reagents using the same procedure as in the flotation tests. Finally, the samples were washed twice with 40 mL distilled water and then dried in a vacuum oven maintained below 40°C. The data were collected and processed using the Thermo Scientific Advantage software. A binding energy of 284.8 eV was adopted as the standard C(1s) binding energy.

### TOF-SIMS Measurements

A crystal sample was prepared for TOF-SIMS measurements, in which the naturally cleaved crystal was polished to a smooth surface in order to meet the requirements for measurement. The sample was soaked in a Pb-BHA complex solution for 3 days before the measurements were carried out. The chemical composition of the scheelite surfaces after adsorption of different agents was determined using an ION-TOF IV instrument (Model 2100 Trift II, Physical Electronics, USA) equipped with a Ga primary ion source. The primary ion beam and acceleration voltage were 25 and 5 keV, respectively. The mass range was set to 200 × 200 μm with 1 mass unit resolution.

## Results and Discussion

### Flotation Experiments and Adsorption Capacity Measurements

**Figure 1:**
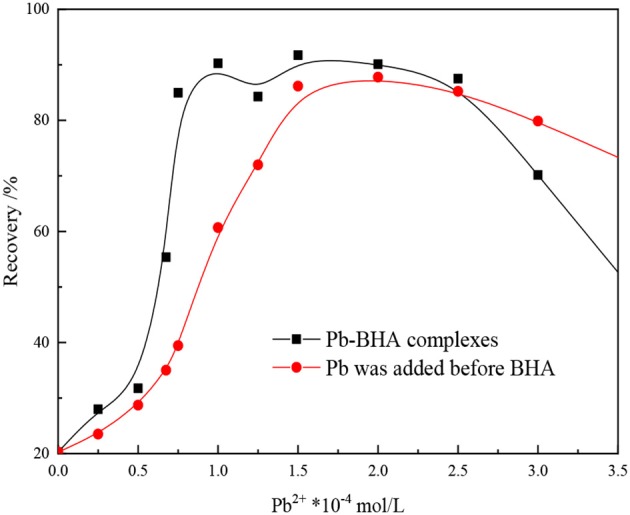
Effect of mode of addition on flotation of scheelite (pH = 8.8 ± 0.2, *C*_BHA_ = 1.5 × 10^−4^ mol/L, C_terpineol_ = 12.5 μL/L).

The effect of dosing mode of the reagents on the floatability of scheelite is shown in Figure 1. As shown in the figure, the way the reagents were added greatly influenced the flotation results, with the mixture of lead ions and BHA within a certain ratio showing a greater collection ability than the two components added sequentially. These results indicate that the Pb-BHA complexes have good collection ability for scheelite flotation and play an important part in conventional lead-activated scheelite flotation.

**Figure 2:**
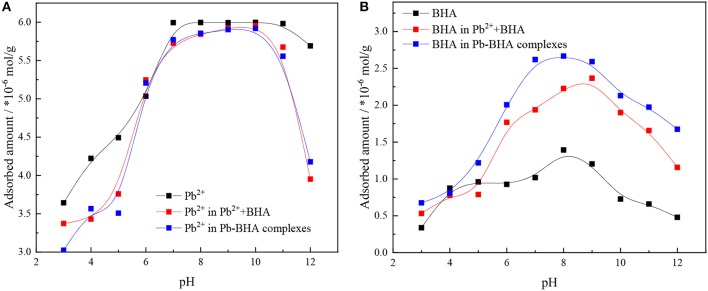
Adsorbed amounts of **(A)** lead ions and **(B)** BHA on scheelite surface vs. pH (*C*_Pb_ = 1.5 × 10^−4^ mol/L, *C*_BHA_ = 1.5 × 10^−4^ mol/L).

Figure 2 shows the adsorption amounts of lead ions and BHA on the mineral surface under activation flotation (shown as Pb^2+^+BHA in figures) and Pb-BHA complex flotation at different pH values. The adsorption capacity was the largest in the pH range 6–10 for both lead ions and BHA. As shown in Figure 2A, there was no significant difference in the amount of lead ion adsorption between the activation flotation and the Pb-BHA complexes flotation, whereas in both cases, the adsorption amount was slightly lower than that of the lead ion alone. As shown in Figure 2B, the adsorption amount of BHA in Pb-BHA complex flotation was higher than that obtained with activation flotation, and both were significantly higher than that of BHA alone; this is consistent with the literature (Tian et al., 2017, 2018a). These results indicate that the addition of lead ions improved the adsorption of BHA on the scheelite surface, and that this improvement was more substantial for Pb-BHA complex flotation.

**Figure 3:**
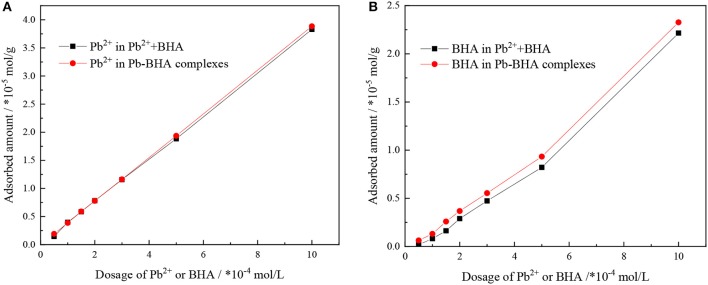
Adsorbed amounts of **(A)** lead ions and **(B)** BHA on scheelite surface at different concentrations of lead ions and BHA (pH = 9.0 ± 0.2, *C*_Pb_:*C*_BHA_ = 1).

The adsorbed amounts of lead ions and BHA on the scheelite surface with different concentrations of lead ions and BHA were measured, as shown in Figure 3, with the molar concentration ratio of lead ions and BHA fixed at 1. The adsorbed amounts of lead ions and BHA on the scheelite surface increased with increasing concentration of lead ions and BHA. As shown in Figure 3A, there was no significant difference in the adsorption of lead ions between activation flotation and Pb-BHA complex flotation, but there was more adsorption of BHA in Pb-BHA complex flotation, consistent with the results shown in Figure 2. The greater amount of BHA adsorbed on the scheelite surface in Pb-BHA complex flotation resulted in a higher flotation recovery than in the case of activation flotation.

**Figure 4:**
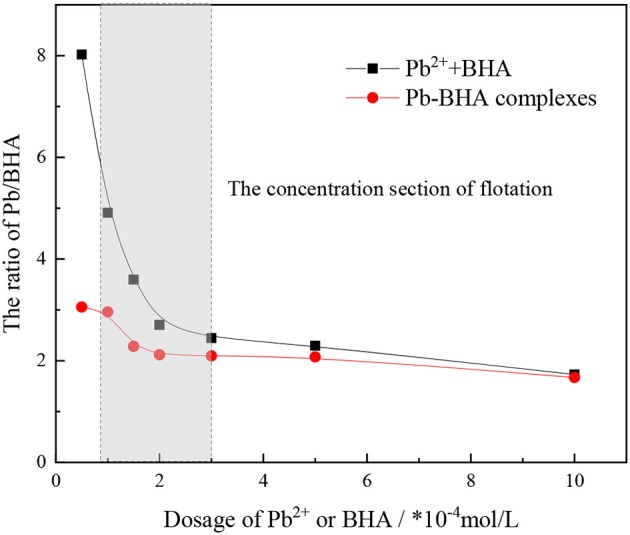
Ratio of BHA and lead ions adsorbed on scheelite surface (pH = 9.0 ± 0.2, *C*_Pb_:*C*_BHA_ = 1).

The ratio of lead ions and BHA adsorbed on the scheelite surface was calculated based on the adsorption amount results, as shown in Figure 4. This ratio changed with increasing concentration of lead ions and BHA, but it eventually became constant. Compared with activation flotation, there was a lower ratio of lead ions and BHA adsorbed on the scheelite surface in Pb-BHA complex flotation, indicating that there may be some difference in the structures of complexes adsorbed in the two cases. In particular, in the concentration section of scheelite flotation from Figure 4 (usually between 1 × 10^−4^ and 3 × 10^−4^ mol/L), the ratio of Pb/BHA was between 2 and 3, which may reflect the average ratio of lead ions to BHA in the molecular structure of complexes adsorbed on the scheelite surface. However, further microscopic measurements are required to confirm this.

### XPS Measurements

Some researchers confirmed that the Pb-BHA complexes existed mainly in the form of Pb-(BHA)_n = 1, 2_ in solution (Han et al., 2017b; Yue et al., 2017; He et al., 2018a), in order to compare the species between Pb-BHA complexes and Pb-BHA complexes adsorbed on mineral surface, the XPS measurements were conducted. Table 1 shows the electron binding energy of the different elements of BHA and Pb-BHA complexes. The binding energies of the O_1s_, N_1s_, and Pb_4f_ atoms underwent a large shift after the BHA interacted with lead ions, indicating that both O and N may bond to Pb to form ring complexes. When BHA is combined with lead ions, the coordination reaction occurs in two forms, as the lead ions can combine either with two oxygen atoms or with one oxygen atom and one nitrogen atom, resulting in an “O, O” five-membered ring or an “N, O” four-membered ring, respectively, and the five-membered ring complexes are the dominating configurations (Fitzpatrick and Mageswaran, 1989; Fernandes et al., 1997; Codd, 2008; Kumar et al., 2018). The significate shift in the electron binding energy of N_1s_ here indicates that the N atoms may have been able to combine with Pb to form “N, O” four-membered rings. Thus, we speculate that both configurations (four- and five-membered rings) may exist in Pb-BHA complexes, it agrees well with the reference (Dianzuo, 1982; Xia et al., 2004; Gupta and Sharma, 2013).

**Table 1:** Electron binding energies of different elements of BHA and Pb-BHA complexes.

**Elements**	**BHA binding energy (eV)**	**Pb(OH)_**2**_**	**Pb-BHA binding energy (eV)**	**ΔE (eV)**
O_1s_	532.27	–	531.18	−1.19
N_1s_	400	–	399.16	−1.05
Pb_4f_	–	138.40	138.70	+0.30

**Figure 5:**
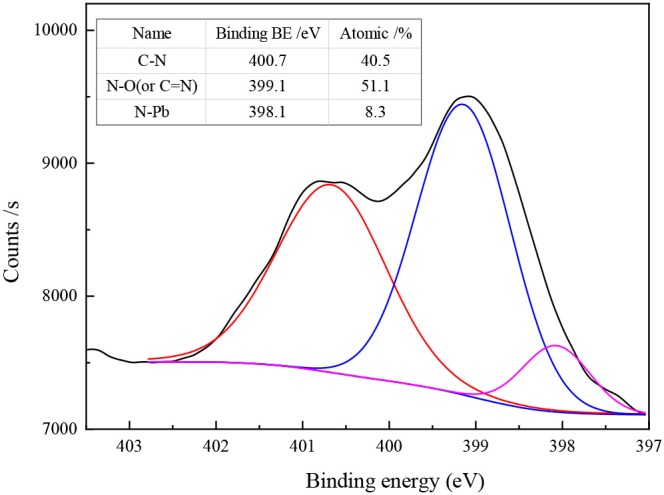
N1s spectra of Pb-BHA complexes.

Figure 5 shows the N_1s_ spectra of Pb-BHA complexes. Four main chemical states of N elements were observed in the Pb-BHA complex: C-N (keto tautomer of hydroxamic acid), C = N (enol tautomer of hydroxamic acid), N-O, and a possible N-Pb coordinate bond (Fitzpatrick and Mageswaran, 1989; Brown et al., 1991, 1996). The binding energies of C-N and N-Pb were about 400 and 398 eV, respectively, but it was difficult to distinguish C = N from N-O because their binding energies were approximately 399 eV (Raole et al., 1987; Jouve et al., 1996; Yuan et al., 2009; Naumkin et al., 2012). The three peaks at 400.7, 399.1, and 398.1 eV (Figure 5) were assigned to C-N, N-O (or C = N), and N-Pb, respectively. The different contents of different forms of N also indicated that the Pb-BHA complexes have multiple configurations.

**Figure 6:**
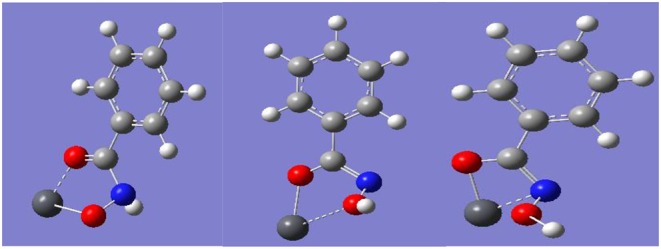
The three possible combinations of a single lead ion and a single BHA.

The different chemical states of N elements represent Pb-BHA complexes with different structures, including complexes with four- and five-membered rings formed by keto and enol tautomers of hydroxamic acid and lead ions (Fitzpatrick and Mageswaran, 1989; Kurzak et al., 1992; Codd, 2008). Three possible combinations of a single lead ion and a single BHA can be inferred from the XPS results (Figure 5), as shown in Figure 6. The -CONHO- group of BHA combines with lead ions to form complexes via coordination, resulting in “O, O” five-membered rings and “N, O” four-membered rings. In the presence of more BHA, a single lead ion can form a complex with two BHAs. Therefore, multiple components of the Pb-BHA complexes exist in the liquid phase, which may be adsorbed on the mineral surface together (Xia et al., 2004; Zhu and Zhu, 2012).

After understanding the XPS spectra and structures of Pb-BHA complexes, we studied the structures of complexes adsorbed on the mineral surface. Table 2 shows the peak binding energies on the scheelite surface before and after treatment with Pb-BHA complexes. The results show that the binding energies of Ca, O, and Pb on the scheelite surface have large positive shifts, the N_1s_ peak have a little negatively shifted, indicating that chemical adsorption plays an important role in the adsorption of Pb-BHA complexes on the surface of scheelite. The large shifts of the O_1s_, Pb_4f_, peaks binding energies indicate that they are active sites for the adsorption process, while the almost negligible shift of the binding energy of N_1s_ indicates that the chemical states of N elements in the complex and on the mineral surface are consistent.

**Table 2:** The peak binding energies on the scheelite surface before and after treatment with Pb-BHA complexes.

**Element**	**Scheelite Peak BE (eV)**	**Pb-BHA complexes Peak BE (eV)**	**Scheelite treated by Pb-BHA complexes**	**ΔE (eV)**
			**Peak BE (eV)**	
C_1S_	284.8	284.8	284.8	0
W_4f_	35.56	–	35.59	+0.03
O_1s_	529.43	531.18	530.44	+1.01
Ca_2p_	346.38	–	346.79	+0.41
Pb_4f_	–	138.70	139.45	+0.75
N_1s_	–	399.16	399.07	−0.09

**Figure 7:**
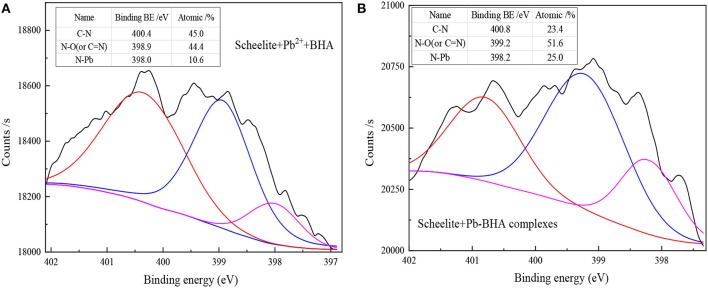
N_1s_ spectra of scheelite after treatment with different reagents: **(A)** lead ions and BHA successively adsorbed on scheelite surface; **(B)** Pb-BHA complexes adsorbed on scheelite surface.

Figure 7 shows the N_1s_ spectra of scheelite after treatment with different dosing modes of the reagents. The results show that the N elements on the scheelite surface also mainly existed in the form of C-N, N-O (or C = N), and N-Pb groups after treatment with Pb^2+^, BHA, and Pb-BHA complexes. This indicates that the species of the component structures of complexes adsorbed on the scheelite surface were similar in two ways. As shown in Figure 7A, when lead ions and BHA were successively adsorbed on the scheelite surface, the relative contents of C-N, N-O (or C = N), and N-Pb groups were 45.0, 44.4, and 10.6%, respectively, compared with 23.4, 51.6, and 25.0%, respectively, for the scheelite surface treated with Pb-BHA complexes (Figure 7B). When lead ions and BHA were successively adsorbed on scheelite surface, there were more component structures with C-N and N-O (or C = N) bonds, whereas more N-O (or C = N) and N-Pb bonds appeared when the scheelite surface was treated with Pb-BHA complexes. This indicated that complexes adsorbed on the scheelite surface had different contents of the various structural components. Overall, according to the XPS results, the components of complexes adsorbed on the scheelite surface had similar species in activation flotation and Pb-BHA complex flotation, but different contents of these species.

### TOF-SIMS Measurements

**Figure 8:**
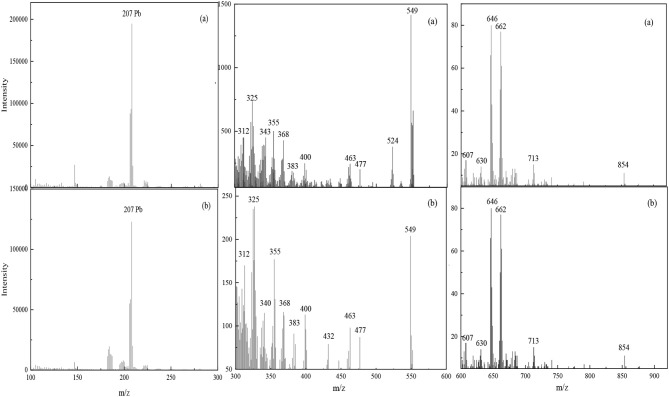
TOF results for Pb-BHA complexes adsorbed on scheelite surface. **(a)** Scheelite treated with Pb^2+^ and BHA sequentially; **(b)** scheelite treated with Pb-BHA complexes.

TOF-SIMS is a highly sensitive analytical technique that can provide chemical characterization of the surfaces of materials (Chehreh Chelgani and Hart, 2014; Xia et al., 2015; Chenakin and Kruse, 2018). TOF-SIMS measurements were obtained to analyze the composition and structure of complexes adsorbed on the scheelite surface in activation flotation and Pb-BHA flotation. Figure 8 shows the TOF-SIMS of a scheelite surface treated with Pb^2+^ and BHA sequentially and scheelite treated with Pb-BHA complexes. High-intensity fragments of all elements were recorded to elucidate the composition and structure of Pb-BHA complexes on the scheelite surface. The peaks detected in the two cases were essentially the same (Figures 8a,b). This was consistent with the XPS results, that is, the species composing the structure of Pb-BHA complexes were similar in activation flotation and Pb-BHA flotation. Thus, the different component contents of complexes adsorbed on scheelite surface were responsible for their different adsorption capacity and flotation behavior.

**Figure 9:**
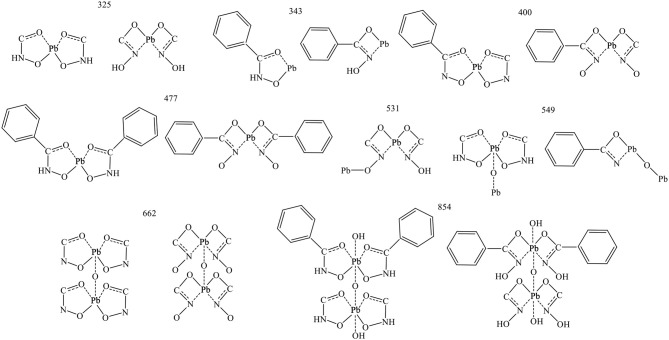
The detected fragments of Pb-BHA complexes adsorbed on scheelite surface.

The composition and structure of the Pb-BHA complexes can be inferred according to the peaks in the detection region, including two forms in which the –CO–NHOH groups bind to lead ions in five- and four-membered rings, respectively. Deduced structure segments detected in the m/z range 100–900 are given in Figure 9 (the five-membered rings of the keto and enol tautomers of BHA are presented in one form). A lead ion could combine with one or two BHA to form monomer complexes; this was the dominant monomer of the complex formed by lead ions and BHA. Furthermore, the two complexes may have been bonded to lead atoms via oxygen atoms, as these two atom types easily combine (Breza and Manová, 1999b; Perera et al., 2001).

**Figure 10:**
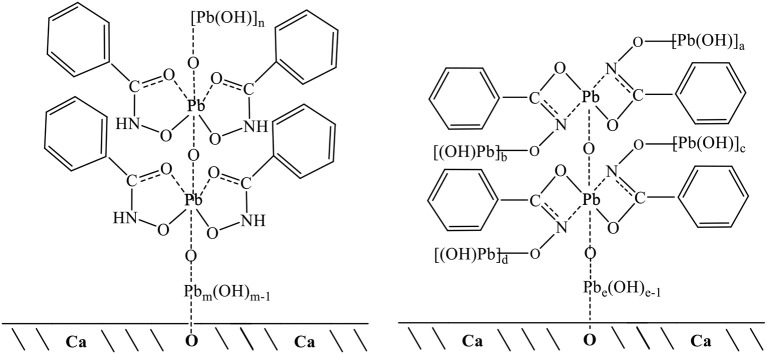
Possible structures of Pb-BHA complexes adsorbed on scheelite surface.

Solution chemical analysis of lead ions has shown that PbOH^+^ and Pb(OH)_2(aq)_ are the lead species in the flotation pH range 7–10 (Han et al., 2018; Tian et al., 2018b), that is, lead ions are in the form of lead hydroxide in solution. Previous research also concluded that polynuclear lead hydroxide species, such as Pb_3_${\left(\text{OH}\right)}_{4}^{2+}$, were formed in the pH range 7–10 (Breza and Manová, 1999a,b; Perera et al., 2001). Thus, lead ions could form polymers of lead hydroxyl in solution, in which lead ions were connected via oxygen atoms (Breza and Manová, 1999b). Here, the XPS and TOF-SIMS results showed that lead ions formed monomer complexes with two BHAs; these Pb-BHA complexes then combined with oxygen atoms. The measurements of adsorption amount indicated that the average ratio of lead ions to BHA in the molecular structures of complexes adsorbed on the scheelite surface was between 2 and 3. Therefore, we speculated that lead hydroxide polymers would bond with oxygen atoms in the complexes. Based on the measurement results, possible structures of Pb-BHA complexes adsorbed on the scheelite surface could be inferred (Figure 10; the five-membered rings of the keto and enol tautomers of BHA are presented in one form). BHA combines with lead ions to form both five- and four-membered rings; both of these configurations may exist on the scheelite surface. Lead ions could form mononuclear complexes with two BHAs, which would then combine with oxygen atoms to form polynuclear complexes. Lead hydroxide polymers with a certain degree of polymerization would bond with oxygen atoms in the complexes. The degree of polymerization of lead hydroxide depends on the pH and the concentration of lead ions in the solution. For the flotation pH and reagent concentrations used in the experiments, we speculated that m+n (or a+b+c+d+e) was 6–10 based on the adsorption amount measurements. At the solid-liquid interface, the functional group of Pb-BHA complexes was lead ions, which could combine with oxygen atoms on the scheelite surface to form an adsorbate. The adsorption of Pb-BHA complexes rendering the surface hydrophobic, and showed a strong collecting ability for scheelite flotation.

## Conclusion

Pb-BHA complexes have good collection ability for scheelite flotation and have important roles in conventional lead-activated scheelite flotation. Adsorption capacity measurements showed that the adsorption amount of BHA in Pb-BHA complex flotation was higher than that in activation flotation, while the results for lead ions were similar. Therefore, more BHA adsorbed on the scheelite surface in Pb-BHA flotation, resulting in higher flotation recovery compared with activation flotation. XPS and TOF-SIMS measurements showed that the species of Pb-BHA complexes were similar in activation flotation and Pb-BHA flotation, but the contents of the three structures of N on the scheelite surface, as determined from the N_1s_ spectra (C-N, C = N, N-O, N-Pb), were different. This was considered to be responsible for the different flotation behaviors. XPS and TOF-SIMS measurements showed that BHA combined with lead ions to form five- and four-membered rings; both of these configurations may exist on the scheelite surface. Lead ions can form monomer complexes with two BHAs, which then combine with oxygen atoms to form polynuclear complexes. Lead hydroxide polymers with a certain degree of polymerization could bond with oxygen atoms in the complexes. The functional group of Pb-BHA complexes was lead ions, which could combine with oxygen atoms on the scheelite surface to form an adsorbate, rendering the surface hydrophobic.

## Data Availability Statement

All datasets generated for this study are included in the manuscript/Supplementary Files.

## Author Contributions

HH and WeiS initiated the research topic. ZW and WenS wrote the manuscript text. YH reviewed and edited the manuscript. RW, YZ, BL, and ZS provided experimental assistance. All authors reviewed the manuscript.

### Conflict of Interest

YZ, BL, and ZS were employed by company BGRIMM Technology Group. The remaining authors declare that the research was conducted in the absence of any commercial or financial relationships that could be construed as a potential conflict of interest.
