# Supplementary material for: Structures of Pb-BHA Complexes Adsorbed on Scheelite Surface
Source: Front Chem. 2019 Sep 24;7:645. doi: 10.3389/fchem.2019.00645 (PMC6798039; doi:10.3389/fchem.2019.00645)
Supplement: Supplementary file 1 [file Data_Sheet_1.PDF]

Supporting information

Structures of Pb-BHA complexes adsorbed on scheelite surface

Zhao Wei[1,2#], Wenjuan Sun[1,2#], Yuehua Hu[1,2], Haisheng Han[1,2*], Wei Sun[1,2*], Ruolin Wang[1,2], Yangge Zhu[3], Bicheng Li[3], Zhenguo Song[3]

1. School of Minerals Processing and Bioengineering, Central South University, Changsha 410083, China

2. Key Laboratory of Hunan Province for Clean and Efficient Utilization of Strategic Calcium-Containing Mineral Resources, Central South University, Changsha 410083, China

3. BGRIMM Technology Group, Beijing, 100160, China

[#]These authors contributed equally to this work

**Corresponding authors: Haisheng Han and Wei Sun Tel: 0086-0731-88830482;*

e-mail: hanhai5086@csu.edu.cn (H. Han), sunmenghu@126.com (W. Sun)

The XRD, FTIR and TGA of Pb-BHA complexes

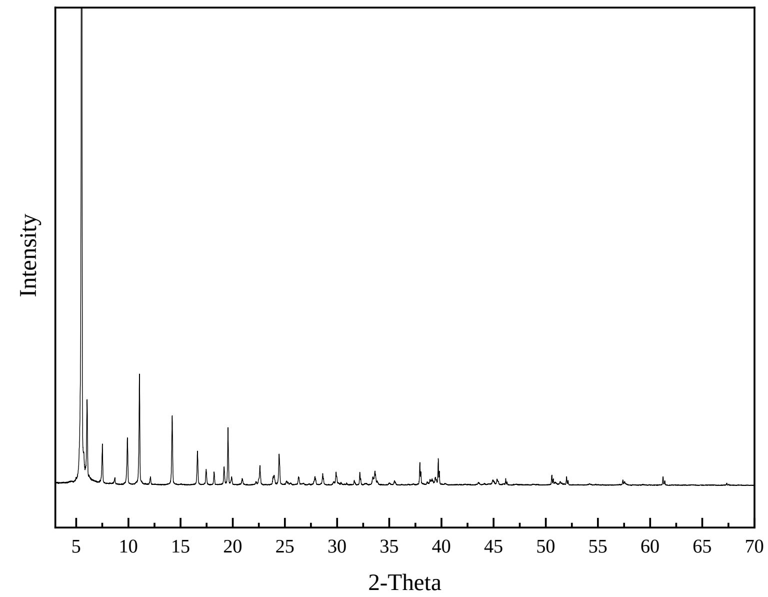

Figure S1 XRD spectrum of Pb-BHA complexes

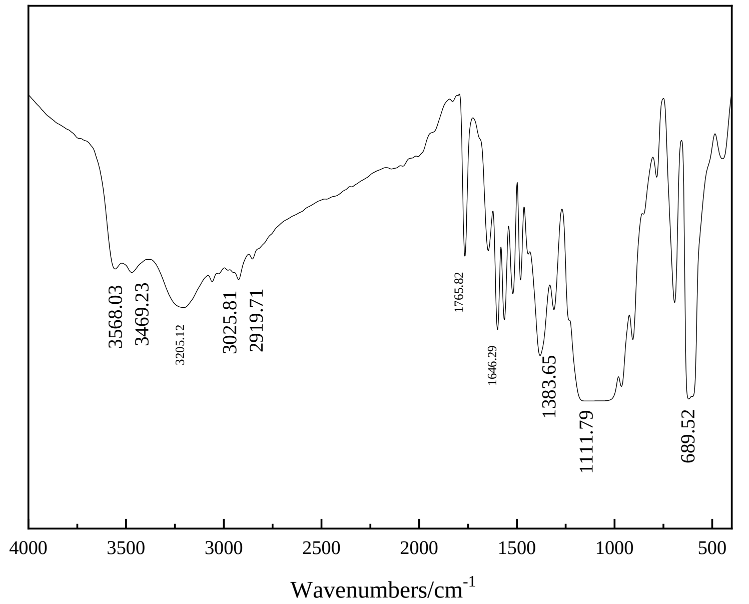

Figure S2 Infrared spectra of Pb-BHA complexes

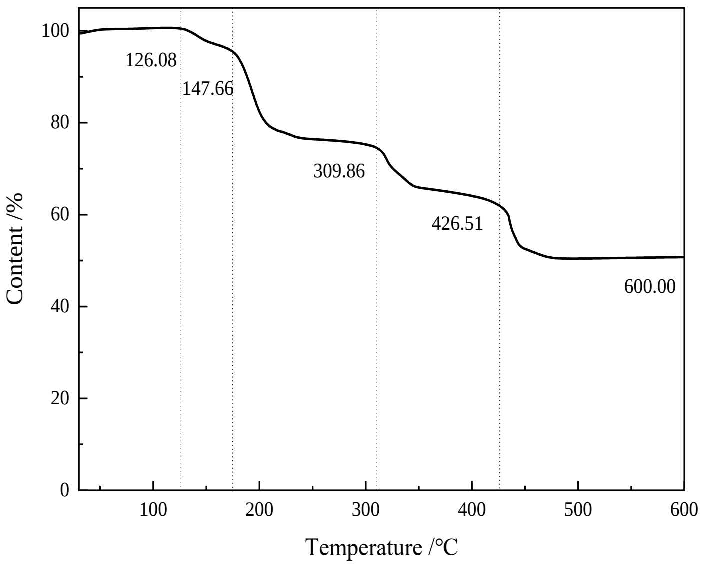

Figure S3 Thermogravimetric analysis of Pb-BHA complexes
